# Pain-related distress in children undergoing air enema reduction for ileocolic intussusception: a proof-of-concept case series

**DOI:** 10.1097/PR9.0000000000001327

**Published:** 2025-09-22

**Authors:** Nachshon Buchshtav, Lea Ohana Sarna Cahan, Neta Cohen, Maor Chavkin, Itai Shavit

**Affiliations:** a Pediatric Emergency Department, Ha'Emek Medical Center, Afula, Israel; b Department of Pediatric Emergency Medicine, Hadassah Medical Center, Jerusalem, Israel; c Faculty of Medicine, Hebrew University of Jerusalem, Jerusalem, Israel; d Pediatric Emergency Department, Tel Aviv Sourasky Medical Center, Tel Aviv, Israel; e Faculty of Medical & Health Sciences, Tel Aviv University, Tel Aviv, Israel; f Hadassah Medical Center, Division of Pediatrics, Ein Kerem, Jerusalem, Israel

**Keywords:** Intussusception, Reduction, Air enema, Child, Pain, Distress

## Abstract

**Introduction:**

Ileocolic intussusception is a leading cause of acute intestinal obstruction among young children. The standard of care treatment is air enema reduction under fluoroscopic guidance. The pain and distress that might be related to this invasive procedure have not been previously investigated.

**Objectives:**

We report on pain-related distress observed in 5 children undergoing air enema reduction for ileocolic intussusception.

**Methods:**

A convenience sample of emergency department patients was assessed using the Face, Legs, Activity, Cry, and Consolability (FLACC) scale conducted at 8 predetermined points throughout the procedure. The staff responsible for the procedure independently recorded their Visual Analog Scale applied by an observer assessment upon procedure completion.

**Results:**

In this sample of children with a median age of 10 months (interquartile range 9–11 months), air enema reduction succeeded on the third attempt in 2 patients, the second attempt in 2 others, and the first attempt in 1 patient. The 4 patients who underwent reduction without sedation or analgesia had first-attempt FLACC scores ranging from 6 to 9, with all maximum scores occurring during air insufflation.

**Conclusion:**

Patients experienced considerable pain-related distress during air enema reduction. These preliminary results represent the first report of pain and distress in children undergoing reduction of intussusception and call for a larger-scale study.

## Abstract 2

Children undergoing air enema reduction of ileocolic intussusception without sedation or analgesia experience considerable pain-related distress.

## 1. Introduction

Ileocolic intussusception, the invagination of the ileum into the colon, is a major cause of acute intestinal obstruction in young children. Abdominal pain is the most frequent symptom and is typically intermittent. Although older patients may present with pain alone, younger patients may initially show lethargy or altered mental status, potentially masking their pain.^13^ The standard-of-care treatment for ileocolic intussusception is air enema reduction under fluoroscopic guidance; without proper treatment, ileocolic intussusception may result in bowel obstruction, mesenteric constriction, and impaired venous blood flow.^17^ In Western countries, annual incidence rates decrease with age: 56/100,000 in infants under 1, 46/100,000 in 2-year-olds, and 38/100,000 in 3-year-olds.^3^ Although air enema reduction is invasive and potentially painful and distressing, there are currently no studies that have evaluated pain and distress in these patients. This lack of research is primarily due to intussusception's low prevalence and a belief that the procedure is not painful.^16^ A recent multinational survey of pediatric radiologists found that clinicians who do not use sedation for ileocolic intussusception reduction primarily attribute this to staffing or logistical constraints, a belief that sedation is unnecessary, or concerns about risks to the child. When asked, “What is the main reason(s) for NOT using sedation or general anesthesia for the reduction of ileocolic intussusception?” respondents cited a range of factors, including lack of immediate access to anesthesiologists or support staff, logistical challenges, concerns about prolonging the procedure, facilities not equipped for anesthesia or sedation, and the absence of established protocols. In addition, some mentioned insufficient evidence supporting the use of sedation or anesthesia as a reason for avoidance.^16^

As a result, treatment involving sedation and/or analgesia is utilized in a minority of cases worldwide.^17^ Importantly, although most intussusception patients undergo air enema reduction without sedative or analgesic treatment, children undergoing colonoscopy, a procedure that also involves air insufflation through the anus, usually undergo the procedure under sedation or a combination of sedation and analgesia.^4,7^

We report on pain-related distress observed in 5 children undergoing air enema reduction for ileocolic intussusception.

## 2. Methods

### 2.1. Study design

A prospective convenience sample of 5 children was conducted in 2 university-affiliated medical centers. The study included emergency department (ED) patients aged 4 to 48 months diagnosed with ileocolic intussusception.

### 2.2. Ethical considerations

The study protocol was approved by the Ethics Committees of the participating hospitals. Caregivers' permission was obtained through an informed consent procedure. The process included obtaining written consent from caregivers that clearly stated that the information provided would be used for a scientific article and that published data would not allow the identification of individual participants. No additional institutional approval was required for publishing case details, as all information used in the case series was derived from observations to which parents had explicitly consented.

### 2.3. Procedure

The procedure of air enema reduction was performed in the radiology suite by a team that included a radiologist, a surgeon, and a nurse. The procedure adheres to recognized professional standards of practice^10,11^: it begins with the rectal insertion of a Foley catheter and the positioning of the infant on the fluoroscopy table. Air insufflation then commences, gradually increasing and is closely monitored with a manometer. The endpoint is the reflux of air into the terminal ileum. If no progress is observed, the pressure increases to 120 mm Hg for up to 2 minutes. The maximum number of attempts is 3, with each reduction attempt not exceeding the 2-minute limit. If a near-complete reduction is achieved, the physician responsible for air insufflation may consider continuing the procedure beyond the 2-minute limit. By following these steps and adhering to the recommended pressure limits, the risk of complications associated with air insufflation can be minimized.^10^ After close consultation with surgeons, a repeated reduction attempt may be performed after a 30-minute to 4-hour delay.^11^

### 2.4. Measures

#### 2.4.1. The face, leg, activity, cry, and consolability

The face, leg, activity, cry, and consolability (FLACC) behavioral scale was the primary outcome measure of the study. The FLACC is an observational assessment tool that measures pain by quantifying scores for 5 separate pain behaviors: facial expression, leg movement, activity, cry, and ability to be consoled. Each behavior is scored 0 to 2, with total scores subsequently ranging from 0 (no pain) to 10 (highest possible pain behavior).^15^ The FLACC scale is one of the most widely utilized behavioral pain assessment scales in pediatric practice. Despite being initially developed and validated for evaluating postoperative pain, the tool has been utilized to assess procedural pain-related distress in various settings, including pediatric emergency medicine.^1,2,6,12,14,18^

#### 2.4.2. The visual analog scale

The Visual Analog Scale applied by an observer (VASobs) was the secondary outcome of the study. The VASobs consists of a horizontal line, 100 mm in length, anchored by word descriptors such as none, annoying, uncomfortable, and worst imaginable pain and/or distress. After providing an explanation of the scale, the investigator asks the observer to mark the line at the point that represents the current state of pain and/or distress.^6^

### 2.5. Data collection

The ED triage nurse notified the study investigators (N.B. or L.O.S.C.) about children potentially eligible for the study. After confirming the diagnosis by ultrasound and before transferring the patient to the radiology suite for the procedure, caregivers were approached by the study investigator who verified inclusion criteria, explained the purpose and design of the study, and obtained informed consent. Immediately after, the investigator recorded demographics, triage pain level, and vital signs. Medications (in ED, preadmission, or during procedure), air insufflation pressures during the procedure, and the number of reduction attempts were recorded by the investigator during the reduction procedure and afterward. Assessments using the FLACC scale were conducted at 8 predetermined points: at baseline (entering radiology suite), when the patient was lying on the fluoroscopy bed when the Foley catheter was inserted into the anus, when air insufflation started, at 50 mm Hg insufflation pressure, at the point of successful reduction, when the Foley catheter was removed, and before the patient left the radiology suite. If more than 1 reduction attempt was required, FLACC scores were recorded at the onset of air insufflation, when the insufflation pressure reached 50 mm Hg, and upon successful reduction. Immediately after the procedure, the nurse, surgeon, and radiologist who participated in the procedure were asked to independently record their assessment of the child's pain and/or distress during the procedure using the VASobs.

### 2.6. Data analysis

Descriptive statistics were generated, including frequencies, medians, and interquartile ranges (IQRs) using StatsDirect statistical software, version 3.3.6 (StatsDirect Ltd., Cheshire, UK).

## 3. Results

**Figure 1.**
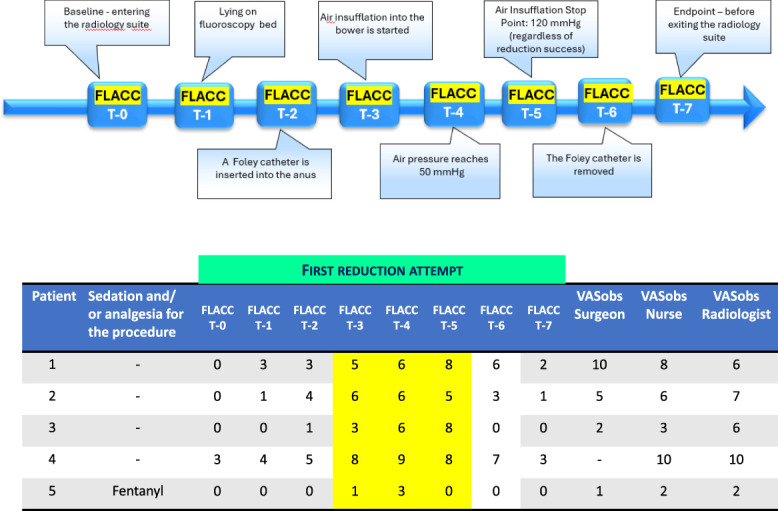
FLACC assessments of the first reduction attempt in 5 children treated with air enema reduction for ileocolic intussusception. The area in the table highlighted in yellow indicates FLACC scores during air insufflation into the bowel. FLACC, face, legs, activity, cry, and consolability; VASobs, Visual Analog Scale applied by an observer.

**Figure 2.**
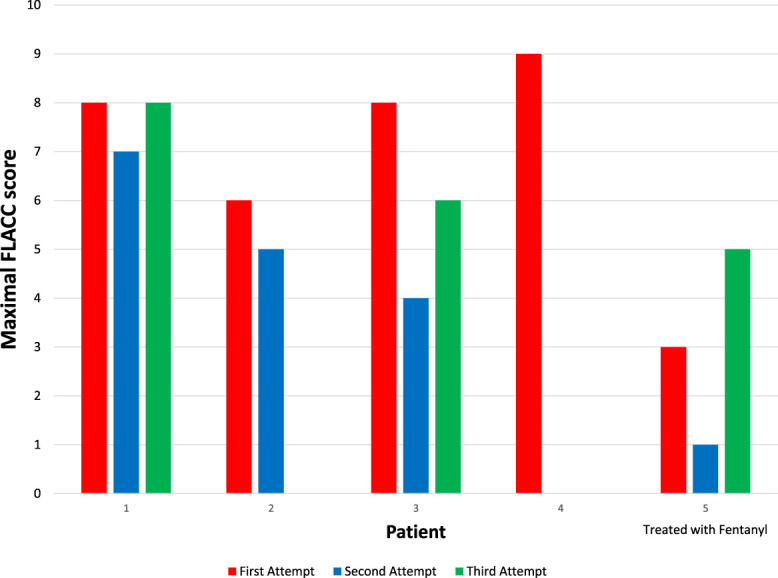
Distribution of maximal FLACC score across reduction attempts for each of the 5 patients. Patients 1 to 4 received neither sedation nor analgesia. FLACC, face, legs, activity, cry, and consolability.

Five previously healthy children (median age 10 months, IQR 9–11) were enrolled between February and August 2024. Four underwent air enema reduction without sedation/analgesia; 1 received fentanyl. The median procedure time was 30 minutes (IQR 20–40 minutes). Reduction succeeded on the third attempt in 2 patients, the second attempt in 2 others, and the first attempt in 1 (Table 1). The 4 patients who underwent reduction without sedative or analgesic medication showed first-attempt FLACC scores ranging from 6 to 9, with all maximal scores occurring during air insufflation (Fig. 1, T3-T5). The distribution of maximal FLACC score across reduction attempts for each of the 5 patients is presented in Figure 2.

**Table 1:** Demographic and clinical characteristics, emergency department data, and procedure details of 5 children treated with pneumatic reduction for ileocolic intussusception.

	Patient 1	Patient 2	Patient 3	Patient 4	Patient 5
Age (mo)	11	7	10	24	9
Gender	Male	Female	Female	Female	Male
Previous intussusception	Yes. At age 3 mo	No	No	No	No
Triage vital signs					
Pain score	0	0	0	3	0
Heart rate (bpm)	120	152	168	128	177
Oxygen saturation (%)	93	96	98	97	97
Blood pressure (mm Hg)	119/76	—	104/70	—	126/76
Rectal temperature (°C)	36.7	—	38.4	36.8	38.0
Main symptoms on admission	Vomiting, paroxysmal pain	Vomiting, drowsiness	Vomiting, irritability, fever	Vomiting, diarrhea	Currant jelly stools
ED analgesic medication	Intravenous paracetamol	Intravenous paracetamol	Intravenous paracetamol	—	Intravenous paracetamol
Analgesic medications for the procedure	—	—	—	—	Intranasal fentanyl 1.5 mcg/kg
Air insufflation pressure at the moment of intussusception reduction (mm Hg)	80	65	70	110	80
No. of reduction attempts	3	2	3	1	3
Total time of the procedure from Foley catheter insertion to the anus until successful reduction (min)	30	20	30	17	40

ED, Emergency Department.

## 4. Discussion

Our preliminary results represent the first report of pain and distress assessment in children undergoing air enema reduction of intussusception. We found that the 4 patients who underwent a reduction of ileocolic intussusception without sedation or analgesia experienced considerable pain-related distress. Importantly, the highest FLACC scores were observed during air insufflation, indicating that trans-anal air introduction may be the procedure's most painful aspect (Fig. 1). These scores remained elevated during subsequent reduction attempts (Fig. 2). The VASobs scores were elevated, indicating that the nurse, radiologist, and surgeon perceived increased pain or distress in these patients. However, these scores should be interpreted cautiously due to the scale's poor interrater reliability and difficulty distinguishing between pain and nonpain-related distress.^6^ The patient administered fentanyl for the reduction showed markedly lower FLACC scores than the other 4 patients. Despite being evaluated in only 1 patient, this observation supports the interpretation that intussusception reduction is associated with significant pain.

Collectively, these findings suggest that pain-related distress levels in this sample were significant, especially in patients who underwent multiple reduction attempts. Importantly, most practices seem to limit reduction attempts to 3 before considering surgical intervention; however, to avoid surgery, some clinicians practice delayed repeated air enema reductions a few hours after initial unsuccessful attempts, potentially increasing pain and distress even further.^11^

Study limitations include the small number of patients studied and the inherent limitations of the study instruments. The FLACC scale was employed due to its widespread use for procedural pain assessment and its reliability and sensitivity in evaluating procedural pain in preverbal children.^6^ However, despite its widespread use for assessing procedural pain in children, the FLACC scale's content validity and feasibility have undergone limited psychometric evaluation.^5^ Although the researchers were experienced Pediatric Emergency Medicine specialists familiar with the FLACC scale, a lack of formal, standardized training is a limitation of this study. A detailed written protocol that included specific scoring criteria and examples for each category was provided to ensure consistency. However, the absence of standardized training and interrater reliability evaluation may have introduced some inconsistency in scoring, which should be considered when interpreting the results. Another potential limitation is the reliance on a single researcher for pain assessment. The ethics committee's prohibition of filming restricted our FLACC evaluations to 1 observer.

In conclusion, our results underscore the risk of significant pain-related distress linked to the reduction of intussusception without sedation and analgesia. A recent multinational survey of pediatric radiologists found that those avoiding sedation or anesthesia cited staffing/logistical issues, perceived unnecessary, or child safety concerns as primary reasons.^16^ Our results constitute the first report on pain and distress observed during intussusception reduction. Importantly, although most health care facilities nowadays perform intussusception reduction without sedation or analgesia, emerging evidence suggests that these interventions can be safely implemented.^8,9,17^

## Disclosures

The authors have no conflict of interest to declare.

## References

[1] Babl FE Crellin D Cheng J Sullivan TP O'Sullivan R Hutchinson A. The use of the faces, legs, activity, cry and consolability scale to assess procedural pain and distress in young children. Pediatr Emerg Care 2012;28:1281–96. 23187981 10.1097/PEC.0b013e3182767d66

[2] Babl FE Goldfinch C Mandrawa C Crellin D O'Sullivan R Donath S. Does nebulized lidocaine reduce the pain and distress of nasogastric tube insertion in young children? A randomized, double-blind, placebo-controlled trial. Pediatrics 2009;123:1548–55. 19482767 10.1542/peds.2008-1897

[3] Buettcher M Baer G Bonhoeffer J Schaad UB Heininger U. Three-year surveillance of intussusception in children in Switzerland. Pediatrics 2007;120:473–80. 17766518 10.1542/peds.2007-0035

[4] Cohen S Glatstein MM Scolnik D Rom L Yaron A Otremski S Ben-Tov A Reif S. Propofol for pediatric colonoscopy: the experience of a large, tertiary care pediatric hospital. Am J Ther 2014;21:509–11. 23567786 10.1097/MJT.0b013e31826a94e9

[5] Crellin DJ Harrison D Santamaria N Babl FE. Systematic review of the Face, Legs, Activity, Cry and Consolability scale for assessing pain in infants and children: is it reliable, valid, and feasible for use? PAIN 2015;156:2132–51. 26207651 10.1097/j.pain.0000000000000305

[6] Crellin DJ Harrison D Santamaria N Babl FE. Comparison of the psychometric properties of the FLACC scale, the MBPS and the observer applied visual Analogue scale used to assess procedural pain. J Pain Res 2021;14:881–92. 33833566 10.2147/JPR.S267839 PMC8020135

[7] Dillon M Brown S Casey W Walsh D Durnin M Abubaker K Drumm B. Colonoscopy under general anesthesia in children. Pediatrics 1998;102:381–3. 9685442 10.1542/peds.102.2.381

[8] Gal M Gamsu S Jacob R Cohen DM Shavit I. Reduction of ileocolic intussusception under sedation or anaesthesia: a systematic review of complications. Arch Dis Child 2022;107:335–40. 34417187 10.1136/archdischild-2021-322706

[9] Hailemariam T Sisay S Mebratu Y Belay F Getinet T Solomon S Belina M Abebe A Hilawi Tewodros B Manyazewal T. Effects of sedatives on radiologic enema reduction in children with ileocolic intussusception: a systematic review and meta-analysis. Eur J Radiol 2024;170:111237. 38039783 10.1016/j.ejrad.2023.111237

[10] Hannon EJ Allan RA Negus AS Murphy F Okoye BO. Air enema reduction of intussusception: a registrar-led, protocol-driven service is safe and effective. Pediatr Surg Int 2013;29:805–9. 23732829 10.1007/s00383-013-3328-2

[11] Kelley-Quon LI Arthur LG Williams RF Goldin AB St Peter SD Beres A Hu YY Renaud EJ Ricca R Slidell MB Taylor A Smith CA Miniati D Sola JE Valusek P Berman L Raval MV Gosain A Dellinger MB Sømme S Downard CD McAteer JP Kawaguchi A. Management of intussusception in children: a systematic review. J Pediatr Surg 2021;56:587–96. 33158508 10.1016/j.jpedsurg.2020.09.055 PMC7920908

[12] Kochman A Howell J Sheridan M Kou M Shelton Ryan EE Lee S Zettersten W Yoder L. Reliability of the faces, legs, activity, cry, and consolability scale in assessing acute pain in the pediatric emergency department. Pediatr Emerg Care 2017;33:14–7. 27977532 10.1097/PEC.0000000000000995

[13] Long B Easter J Koyfman A. High risk and low incidence diseases: pediatric intussusception. Am J Emerg Med 2025;91:37–45. 39987626 10.1016/j.ajem.2025.02.020

[14] Lorente S Romero A Martínez M Martínez-Mejías A. Effectiveness of procedural sedation and analgesia in pediatric emergencies. A cross-sectional study. J Emerg Nurs 2023;49:75–85. 36376128 10.1016/j.jen.2022.10.004

[15] Merkel SI Voepel-Lewis T Shayevitz JR Malviya S. The FLACC: a behavioral scale for scoring postoperative pain in young children. Pediatr Nurs 1997;23:293–7. 9220806

[16] Meshaka R Müller LSO Stafrace S Abella SF Sofia C Calder A Petit P Perucca G. Intussusception reduction methods in daily practice-a survey by the European Society of Paediatric Radiology Abdominal Imaging Taskforce. Pediatr Radiol 2023;54:571–84. 37993547 10.1007/s00247-023-05798-0

[17] Poonai N Cohen DM MacDowell D Mistry RD Mintegi S Craig S Roland D Miller M Shavit I Patel N Gutierrez C Roben E Pruitt C Quayle KS Rolin A Kornfeld D Davis J Grupp-Phelan J Bogie A Territo H Hershman E Kolbergs J Stanton V Sheedy S Forester S Binham L Dell'Era L Tornesello A Wang Y Nager A Heyming T Burns R Trehan I Lipshaw M Sulton C Li J Ojo A Kelly S Thornton M Caperell K Amoni I Abrams A Duong M Wassem M Davis A Gravel J Doyon Trottier E Bar Am N Thompson G Sabhaney V Meckler G Jain R Ali S Bressan S Zangardi T Villa G Giacalone M Seiler M Sahyoun C Romano F Bognar Z Hajosi-Kalcakosz S Amir L Hachimi-Idrissi S Pucuka Z Zviedre A Zeltiņa E Phillips N Borland M O'Brien S Marchant J Kochar A George S Pennington V Lyttle M Browning J McLoughlin A Hartshorn S Urooj C Johnston L Walton E Subrahmanyam Puthucode D Peacock P Conroy J Marañon R Garcia S Cahís N Cámara-Otegui A Gomez A Carbonero M Angelats-Romero C Yock-Corrales A Hualde G Spigariol F Donas A Gübeli Linné C Rocchi A Pedrazzini A Cozzi G Barbi D Baggio L La Fauci G Mauro A Steimle M Buonsenso D Ugalde I Nieva G Harper C Sforzi I Jain S. Sedation and analgesia for reduction of pediatric ileocolic intussusception. JAMA Netw Open 2023;6:e2317200. 37285152 10.1001/jamanetworkopen.2023.17200 PMC10248743

[18] Scribner-O'Pray M Taylor ED Krause E Nickel A Bergmann KR. Factors associated with low procedural pain scores among 1- to 5-year-old patients undergoing facial laceration repair. Pediatr Emerg Care 2023;39:135–41. 35608526 10.1097/PEC.0000000000002744

